# A Strange Calculus

**DOI:** 10.4269/ajtmh.18-0246

**Published:** 2018-10

**Authors:** Timothy S. Laux

**Affiliations:** Jan Swasthya Sahyog/People’s Health Support Group, Bilaspur, India; Columbia University Medical Center, New York, New York

In early 2018, a young woman arrived to our emergency department. She was obviously ill. She had been diagnosed with tuberculosis (TB) 4 months previously after a lengthy illness and was already completing her fourth month of TB medications without improvement.

She needed a bed.

Our hospital, like many others in low- and middle-income countries, is plagued by a constant bed shortage. My Indian colleagues often refer to this as “the common problem”; it is so pervasive in daily work. Low- and middle-income countries, in general, lack sufficient inpatient beds.^1,2^ For both outpatient care and elective surgeries, we use a first-come, first-served system. If any of those patients need admission, we strive to prioritize them. Thereafter, the admission algorithm becomes much less clear. We try to make decisions about patient admissions without considering the lack of beds. Unfortunately, these shortages make that task very difficult. This means that one of two possibilities usually awaits the emergency patient—like her—who comes to the hospital when a bed is not available. She is either 1) referred to another hospital where there is no guarantee of a bed or 2) we attempt to stabilize her enough that she can wait in the first-come, first-served queue. Either way, you must be very sick to be allowed a bed in emergency for more than a few minutes. Those who are not sick enough are quietly sent to the outpatient department (OPD) queue.

The leading differential diagnosis on the first-year resident’s list was lupus. I agreed. Lupus nephritis—where lupus relentlessly attacks the kidneys—is a medical emergency. We carefully questioned, examined, and performed the necessary laboratory procedures to feel comfortable that she did not yet have that problem. I remember peering at her urine with the laboratory technicians, squinting through my thick lenses at the dark slide under the microscope as we searched for any of the telltale features of lupus nephritis.^3^ There were none at that time.

In her case, we chose the second option for emergency patients—close OPD follow-up. The confirmatory testing for lupus often takes a week to return, so we scheduled her follow-up for 10 days later (to guarantee that she did not have to wait for her result on returning). We also asked her to come with the paperwork from when her TB was diagnosed. Warning signs and reasons to return to the hospital were discussed. She and her family agreed with this plan. Although the best-case scenario would have been an admission, I recall thinking our plan was a reasonable second best option, given our limitations.

There are no shortages of emergencies in rural, northern Chhattisgarh. However, a functioning public health system for emergencies assumes a certain number of emergency departments. With sufficient numbers, the goal is for the sick to be able to arrive before death or permanent disability occurs. This assumption can be a stretch in the richest of settings with quaternary-care hospitals, trained paramedics, and functioning ambulances. In rural India—the largest part of a nation of 1.3 billion that spends only 1–2% of gross domestic product on health care^4,5^—prehospital care does not exist. Unless the emergency happens very close to the hospital, the patient cannot reach the hospital in time. As such, our emergency department tends to care for patients whose illnesses are long term but acutely out of control.

This reality leads to a very strange calculus for doctors, and perhaps the most difficult and heart-rending aspect of our work in rural India—adjudicating what is sick enough to be allowed to stay in the emergency department to receive care before admission or transfer and what must be returned to the lengthy queue. It should be noted that this is not the reality throughout India. It is only our special circumstances—rural location, indigenous population that feels very uncomfortable in large, public hospitals, an anecdotal belief among our patient that outcomes are often worse in government hospitals and a complete inability to access the high-cost private health-care sector—that maintains this difficult calculus.

We are asked to place the unassailably sick into two unnatural categories—those who are not so sick and can live a few more days with a long-term problem and those whose chronic disease has gone so totally awry that immediate medical care is necessary. This is not a calculus that any medical practitioner is trained in. It may sound easy but it is not. Only extreme circumstances can force us to participate in it. It is inhumane for both the doctor and the patients.

It is also an imperfect calculus. Nine days later, that same young woman was rushed back into the emergency department, looking so much worse that I did not initially recognize her. Her family reported that shortly after returning home, she had begun to produce dark red urine and became more swollen. The family had thought of returning but drew false comfort from her upcoming appointment and decided to stay home. They were only returning on the ninth day because of that appointment but, on arriving, they felt like they should go straight to the emergency department. Despite our emergency department’s constant returning of the not quite sick enough to the queue, we did not hesitate to give her a bed.

In those intervening days, she had developed lupus nephritis. Her kidneys were under assault. Now her urine looked like a picture from a medical textbook, full of white blood cell casts.^3^ The diagnostic test for lupus we sent 9 days previously had returned. It was unequivocally positive. She was directly admitted to the intensive care unit and, after a hasty discussion about how lupus nephritis medications might make her infertile, she was started on aggressive therapy. Bravo for our diagnosis and bravo to her family for exactly following the directions about her medical care.

She was dead less than 48 hours later.

This is profoundly upsetting on many levels as the physician who sent her home just 9 days previously. At that time, I was fairly confident of her diagnosis and slightly less confident of her clinical stability. Although the global health practitioner is well versed in practicing medicine at its limits and making the best of a bad situation—here, the permanent bed shortage in rural Chhattisgarh—this bad situation could not have turned out worse.

Nine days earlier, just about anywhere in a high-income country (HIC), she would have been admitted. If our bed situation had somehow been less tight, I would have rapidly admitted her, too. But, without a bed available, the question became just how much of an emergency this illness was. Did she require immediate transfer or could she safely be followed up in the OPD? I determined she could last until her confirmatory test returned. She did by 2 days.

Similarly disquieting is the knowledge that if she had shown up 1 month previously, my calculus would have been survivable and she would have likely left the hospital alive and improving. She did, in fact, show up somewhere and faithfully accepted their diagnosis and took their medications for four long months. I chose to hold off on any steroids or other immunosuppressive therapies after her first visit because I simply could not be sure about her possible TB diagnosis. Maybe it was clear cut 4 months ago and she had greatly improved despite her denials to that effect. Steroids can potentially worsen some forms of TB. Rural Indians often have multiple, separate, severe diseases.

The challenge of global health work is often portrayed as one of an intimate contact with broken public health systems, exhausted and under compensated local staff, and preventable death.^6,7^ These realities cannot be denied. Nonetheless, we fail to capture the full range of skills that any health-care practitioner is asked to deploy in low resource settings when we only consider such “big picture” issues. Many of the biggest hurdles and aspects of global health work that are hardest to communicate to our HIC colleagues—like this bad calculus—are things for which no practitioner is trained but which are an inescapable daily reality.
